# Working conditions, authorizations, mental health, and job satisfaction of physician assistants in Germany

**DOI:** 10.3389/fpubh.2023.1082463

**Published:** 2023-02-23

**Authors:** Yvonne Treusch, Luis Möckel, Karin Kohlstedt

**Affiliations:** ^1^Department of Health and Social Affairs, HSD University of Applied Sciences, Cologne, Germany; ^2^IU Internationale Hochschule GmbH, University of Applied Sciences, Düsseldorf, Germany

**Keywords:** physician assistant, job satisfaction, depression, anxiety, stress, responsibility, authorization, burnout

## Abstract

**Objective:**

This study explores associations among the overall and facet-specific job satisfaction, work-related factors, responsibilities, and mental health of physician assistants (PAs) in Germany to identify factors that prolong the lifetime and wellbeing of PAs in practice and to counteract the shortage of healthcare staff.

**Methods:**

An online survey comprising sociodemographic and work-related items, items from the short questionnaire of general and facet-specific job satisfaction (KAFA), and the Depression, Anxiety, and Stress Scale (DASS-21) were distributed to PAs working in Germany in 2021 (cross-sectional survey design). Descriptive statistics, DASS-21 subscale score analysis, *t*-test, ANOVA, or Kruskal–Wallis test was used.

**Results:**

PAs (*n* = 169) were working mainly in surgery (23.2%), internal medicine (20.3%), or orthopedics and trauma surgery (17.5%), whereas only a few PAs were working in emergency care, geriatrics, neurology, or oncology. They were responsible for a broad spectrum of medical activities depending on the practice setting. PAs working in emergency care claimed to be the most empowered, followed by PAs in orthopedics and surgery. Almost all PAs carried out documentation, anamnesis, and diagnostic services. Although almost all PAs rated their overall job satisfaction as good, satisfactory, or pleasant (91.6%), single facets of job satisfaction were rated differently. Colleagues and supervisors were assessed very positively, whereas payment and professional activities were rated rather average and development opportunities even worse. PAs working in oncology demonstrated the highest overall job satisfaction, followed by PAs working in geriatrics and emergency care. Overall job satisfaction was significantly negatively associated with depression, anxiety, and stress scores (*p* ≤ 0.001, *p* ≤ 0.05, and *p* ≤ 0.05, respectively). Particularly, female gender, having an urban residence, and PAs working in oncology demonstrated significantly increased anxiety scores. Moreover, depression scores of PAs working in oncology or neurology or with a low net income exceeded critical cutoff values.

**Conclusion:**

Interventions aimed at removing the significant negative correlation among job satisfaction, depression, anxiety, and stress scores are needed. To retain PAs in their jobs, salary, autonomy, and development opportunities should be improved and prevention programs for anxiety and depression should be offered. Remarkably, PAs' overall good job satisfaction was mainly determined by good evaluations of supervisors and colleagues.

## Introduction

The first physician assistant (PA) program in Germany started in 2005 in accordance with the trend in other countries of educating PAs to avoid the imbalance between the demand and supply for healthcare (1). PAs are initiated to relieve the healthcare system and its employees by routinely taking on delegable tasks from physicians. Depending on the work experience of PAs and the focus of the practice of the supervising and delegating physician, the tasks and responsibilities of PAs can vary (1, 2). There are currently at least 22 universities of applied sciences in Germany that offer a degree in Physician Assistance (3), and some of them have joined forces to form the German University Association for Physician Assistants (Deutscher Hochschulverband Physician Assistant e.V., DHPA e.V.) (4). A survey of former students of universities belonging to the DHPA showed that PAs were highly satisfied with their choice of career and being fully employed (5). However, PAs are still new in Germany, and their acceptance is slowly being tested in practice (2). Although more than 1,000 PAs are now practicing their profession (3), very little is known about their everyday life and scope of practice in Germany, empowerment, mental health, and job satisfaction.

Job satisfaction is an important concept in occupational medicine and is positively correlated with health and wellbeing (6). A high level of job satisfaction has a positive effect on work performance, health, and behavior at work, and satisfied employees tend to be more productive and creative (7–9). Moreover, employees with greater job satisfaction are less likely to leave their jobs than those who are dissatisfied (6, 10, 11). The general, overall job satisfaction is a multidimensional concept consisting of many components and defined as the employee's overall attitude to the work since employees balance their job satisfaction or dissatisfaction to different parts of the job (facets) and finally form an overall conclusion about the job (6, 7). There were several tools discussed and tested to determine job satisfaction, of which the job descriptive index is one of the best established (6). It includes five facets of job satisfaction: employment, salary, promotion opportunities, supervision, and coworkers. These five facets are included in many methods of surveying job satisfaction (7). Understanding PAs' job satisfaction is important for recruiting and retaining those professionals.

In Germany, the overall shortage of registered healthcare staff is high (12), and the sickness absence value in the healthcare industry is at the top and clearly above the average of all industries in Germany (13), leading to an increased workload, job stress, job dissatisfaction, and even burnout of all healthcare professionals (14, 15). Indeed, several studies confirmed that increased job stress negatively affects job satisfaction and wellbeing and suggested that job dissatisfaction may lead to symptoms of burnout (16–21). It was believed that job satisfaction with one's current position may be a protective factor against burnout (22).

Burnout as a work-related stress syndrome resulted from chronic exposure to job stress and is common among healthcare workers (23, 24). It is characterized by the dimensions of emotional exhaustion, depersonalization, and lack of personal accomplishment (22). The physical and psychological exhaustion associated with different types of burnout were reflected in symptoms of depression, anxiety, and stress (25), and the Depression, Anxiety, and Stress Scale-21 (DASS-21) questionnaire was suggested to be an excellent tool for measuring depression, hyperarousal, and tension in the clinical and non-clinical groups (26, 27). In addition, assessments of facets of job satisfaction were good predictors for less exhaustion, less depersonalization, lack of empathy, and higher personal accomplishment (16). Thus, information obtained by measuring facets of job satisfaction or dissatisfaction can help identify stress indicators and causes of psychological stress and derive appropriate intervention measures (7).

Burnout may lead to broken relationships, drug use (about 25% increased odds of alcohol abuse/dependence), and a nearly doubled risk of suicidal ideation and depression (23, 28, 29). Indeed, a meta-analysis revealed that physicians are an at-risk of suicide profession with a global standardized mortality rate (i.e., the ratio between the observed and expected number of death) by suicides of 1.44 (30). Moreover, a cross-sectional study on Austrian physicians revealed that the odds ratio of suffering from major depression was 2.99 for physicians with mild, 10.14 for physicians with moderate, and 46.84 for physicians with severe burnout in comparison to physicians unaffected by burnout (31). The effects of burnout on medical care workers may result in medical errors and reduced quality of patient care (23, 28, 29). A systematic review including 46 studies described a significant association between burnout and patient safety or burnout and error (32). Moreover, a recent cross-sectional nationwide survey of German prehospital emergency medical services workers demonstrated that burnout is significantly associated with safety outcomes (33). The authors analyzed emergency medical service workers with a low, average, or high degree of emotional exhaustion and depersonalization and demonstrated that the percentage of participants with a high degree of emotional exhaustion and depersonalization was greater for those who reported injuries or errors and adverse events (e.g., 50% of the participants who reported injuries and 44% of those reported errors and adverse events exert a high degree of depersonalization). Moreover, a recent study with physicians and nurses in Germany demonstrated that a shorter disease-related length of stay in the hospital was associated with a lower risk of physician burnout (34). Prevention of burnout and promotion of engagement will be valuable for healthcare teams and society's overall health (35).

A systematic review of the prevalence of burnout among physicians including 182 studies involving 109,628 individuals in 45 countries published between 1991 and 2018 revealed that 67.0% of the studies reported an increased prevalence of overall burnout (36). Moreover, several studies and surveys from recent years demonstrated that medical health workers have an increased prevalence of anxiety, depression, and burnout in comparison to peers in non-medical careers (14, 37, 38). Studies from the U.S. have demonstrated that PAs especially are working in areas with high burnout prevalence such as emergency medicine, primary care, hospice and palliative care, and oncology (35) and appear to develop burnout at levels similar to their physician colleagues with rates of burnout between 34 and 64% (39, 40). In Germany, prevalence rates of burnout among medical staff are reported to be equally high: about 35–38% for general practitioners (14, 41) and up to 40% for a high degree of burnout within the emergency medical staff (33). In Germany, no explicit data on stress, anxiety, depression, or burnout among physician assistants are available, but poor psychosocial working conditions and a negative influence of working conditions on the quality of care were reported for PAs in Germany (42).

This study aimed to report the overall and facet-specific job satisfaction and mental health of PAs in Germany and link the findings to sociodemographic or work-related factors. Identification of factors that prolong the lifetime and wellbeing of PAs in practice can help to derive appropriate intervention measures to avoid losing highly qualified PAs and shortage of healthcare staff and to sustain patient safety and care. Moreover, the results provide an overview of PA working areas and authorization in Germany to represent the job profile.

## Materials and methods

### Study design

This study was a cross-sectional survey study conducted from May to July 2020 using the online survey tool SoSci Survey (43). The link to the survey was distributed to PAs working in Germany through the snowball system and by the German University Association for Physician Assistants (DHPA, Deutscher Hochschulverband Physician Assistant e.V.). For snowball sampling, the link to the survey was sent to the working email address of the PA network of the authors and DHPA. In addition, participating PAs were asked to further distribute the link within their network of colleagues.

Participation in the survey was voluntary, and the study participants could have ended the survey at any time and did not belong to a vulnerable group. The data were handled in accordance with the local data protection regulations and were not shared with a third party. Study participants did not receive any compensation for their participation in the survey study. The study was approved by the ethics committee of the HSD University of Applied Sciences, Germany (BEth_54_222). Study participants had no time limit to answer the questionnaire and the time for answering the questionnaire varied between 5 and 8 min.

### Questionnaire

The survey consisted of questions assessing sociodemographic (age, gender, family status, and region) and work-related items (net income per month, medical working area, and responsibilities), items from the short questionnaire of general and facet-specific job satisfaction (KAFA, Kurzfragebogen zur Erfassung von Allgemeiner und Facettenspezifischer Arbeitszufriedenheit) by Haarhaus (7), and items from the German version of the Depression, Anxiety, and Stress Scale (DASS-21) (44, 45).

The KAFA was used to evaluate the job satisfaction of study participants. It is based on the Job Descriptive Index (46) and validated for a German sample with satisfactory psychometric properties (7). It included both general and facet-specific job satisfaction in six items with a total of 30 questions related to the work itself, coworkers, promotions, pay, and supervision. In the actual version of the KAFA, each question had to be rated with a 5-point Likert scale. To shorten the questionnaire, items of the KAFA were reduced without changing the original items. Only one answer per item could be selected.

The DASS-21 was used to monitor depression, anxiety, and stress of study participants. It is a 21-item questionnaire with three 7-item subscales. Each item is scored on a 4-point scale [ranging from never (0) to always (3)]. Subscale scores were calculated as the sum of the responses to the seven items from each subscale multiplied by 2 to get scores equivalent to the 42-item full DASS. The cutoff scores for DASS-21 were taken from Lovibond and Lovibond (44): depression (normal 0–9, mild 10–13, moderate 14–20, severe 21–27, extremely severe 28+), anxiety (normal 0–7, mild 8–9, moderate 10–14, severe 15–19, extremely severe 20+), and stress (normal 0–14, mild 15–18, moderate 19–25, severe 26–33, extremely severe 34). For the cutoff values of 10 for depression, 8 for anxiety, or 15 for stress, an increased expression of these characteristics can be assumed.

### Statistical analysis

Characteristics of study participants are presented as mean with standard deviation (SD) for continuous data or proportions for categorical data. The responsibilities of study participants in the medical working area are shown in proportions.

For the evaluation of the information on job satisfaction, the percentage of study participants per item answer was calculated.

For the analysis of DASS-21 subscale scores, the respective items for each subscale were summed up and multiplied by 2, to receive values equivalent to the full version of the DASS-21 (44, 45). To identify differences in DASS-21 subscale scores by gender, region, working unit, responsibilities, how often these responsibilities were carried out, net income per month, and overall job satisfaction, mean values and corresponding SD were calculated and analyzed using *t*-test and ANOVA. If the requirements for ANOVA were not fulfilled, the Kruskal–Wallis test was used. Statistical analysis was performed using the JASP software package (47), and a *p*-value of ≤ 0.05 was considered statistically significant.

## Results

### Characteristics of study participants

A total of 169 PAs [estimated 17% of all German PAs (5)] were included in the final analysis. The mean age of study participants was 30.3 (SD 8.0) years, and 84.0% were women (Table 1). The majority of the participants were single (40.4%), 36.1% were living with partners, and 22.5% were married. A total of 52.7% stated to have a net income of 2,000–2,499 EUR per month, 31.9% ≥2,500 EUR per month, and 10.1% 1,500–1,999 EUR per month. Most of the study participants were working in urban regions.

**Table 1:** Characteristics of study participants.

**Characteristics**	***n* = 169**
Age—years (SD)	30.3 (8.0)
**Gender**	
Men	16.0%
Women	84.0%
**Family status**	
Single	40.4%
In partnership	36.1%
Married	22.5%
Divorced	2.4%
Widowed	0.0%
**Net income per month**	
≤ 999 EUR	1.8%
1,000–1,499 EUR	3.6%
1,500–1,999 EUR	10.1%
2,000–2,499 EUR	52.7%
≥2,500 EUR	32.0%
**Region**	
Urban	62.7%
Rural	37.3%

### Authorization and responsibilities of PAs working in different medical areas

Assessing the authorization of PAs depending on the medical field of work (except oncology, for which no data were available) revealed that documentation, anamnesis, and diagnostic services were job responsibilities of nearly all PAs, whereas other job responsibilities differ (Table 2). Surgery participation and, to a lesser extent, after-care were the main job responsibilities of PAs working in surgery or emergency care. It was interesting to note that most of the PAs in orthopedics and trauma surgery were responsible for team coordination (76%). In internal medicine and neurology, a high percentage of PAs (79.3 or 71.4%, respectively) were responsible for patient information. Intervention/counseling, treatment suggestions, as well as medical reporting or diagnostic analysis, were the main responsibilities of PAs in emergency care and, to a lesser extent also, of PAs in geriatrics and surgery.

**Table 2:** Authorization of German physician assistants (PAs) in total and depending on the medical working area.

	**Medical area**								
	**Surgery**	**Internal medicine**	**Orthopedics**	**Emergency**	**Geriatrics**	**Neurology**	**Oncology**	**Others**	**Total**
Percentage of total PAs	**23.1**	**20.3**	**17.5**	**7.7**	**5.6**	**4.9**	**2.8**	**18.2**	**100**
**Authorization**									
Documentation	94.0	86.2	92.0	100.0	100.0	100.0	n/a	90.0	**94.6**
Anamnesis	84.9	82.8	76.0	100.0	87.5	85.7	n/a	75.0	**84.6**
Diagnostic services	60.6	75.9	64.0	72.7	87.5	85.7	n/a	72.5	**74.1**
Intervention/counseling	72.7	62.1	64.0	100.0	75.0	57.1	n/a	70.0	**71.6**
Treatment suggestions	42.4	65.5	68.0	100.0	75.0	28.6	n/a	62.5	**63.1**
Medical report/diagnostic analysis	57.6	65,52	40.0	91.0	75.0	42.9	n/a	52.5	**60.6**
Patient information	51.5	79.3	52.0	54.6	50.0	71.4	n/a	62.5	**60.2**
Team coordination	42.4	37.9	76.0	45.5	50.0	28.6	n/a	50.0	**47.2**
Prevention and instruction	45.5	37.9	44.0	54.6	25.0	57.1	n/a	40.0	**43.4**
Surgery participation	84.9	10.4	92.0	27.3	0.0	14.3	n/a	52.5	**40.2**
After-care	45.5	10.4	60.0	27.3	12.5	28.6	n/a	57.5	**34.5**
Other responsibilities	3.0	24.1	16.0	18.2	25.0	14.3	n/a	27.5	**18.3**
Nursing activities	15.2	6.9	20.0	36.4	0.0	0.0	n/a	7.5	**12.3**
Telemedical care	12.1	3.5	24.0	18.2	0.0	0.0	n/a	12.5	**10.0**
Home visits	0.00	0.00	0.0	0.0	0.0	0.0	n/a	0.0	**0.0**
Average of percent authorization	**47.5**	**43.2**	**52.5**	**56.4**	**44.2**	**41.0**	**n/a**	**48.8**	**47.7**

Results were demonstrated as a percentage of total PAs (n = 169; bold) or as a percentage of PAs working in different medical areas. To identify the medical area with the most authorized PAs, the average percent authorization was calculated per each subgroup of PAs (average percent authorization; n/a, not available).

To be able to give an estimate of which profession has the most powers, the percentages of all powers of a subgroup of PAs were added up and the average percentage was calculated. The average powers are highest in the group of PAs working in emergency care followed by PAs in orthopedics and surgery.

### Job satisfaction

**Figure 1:**
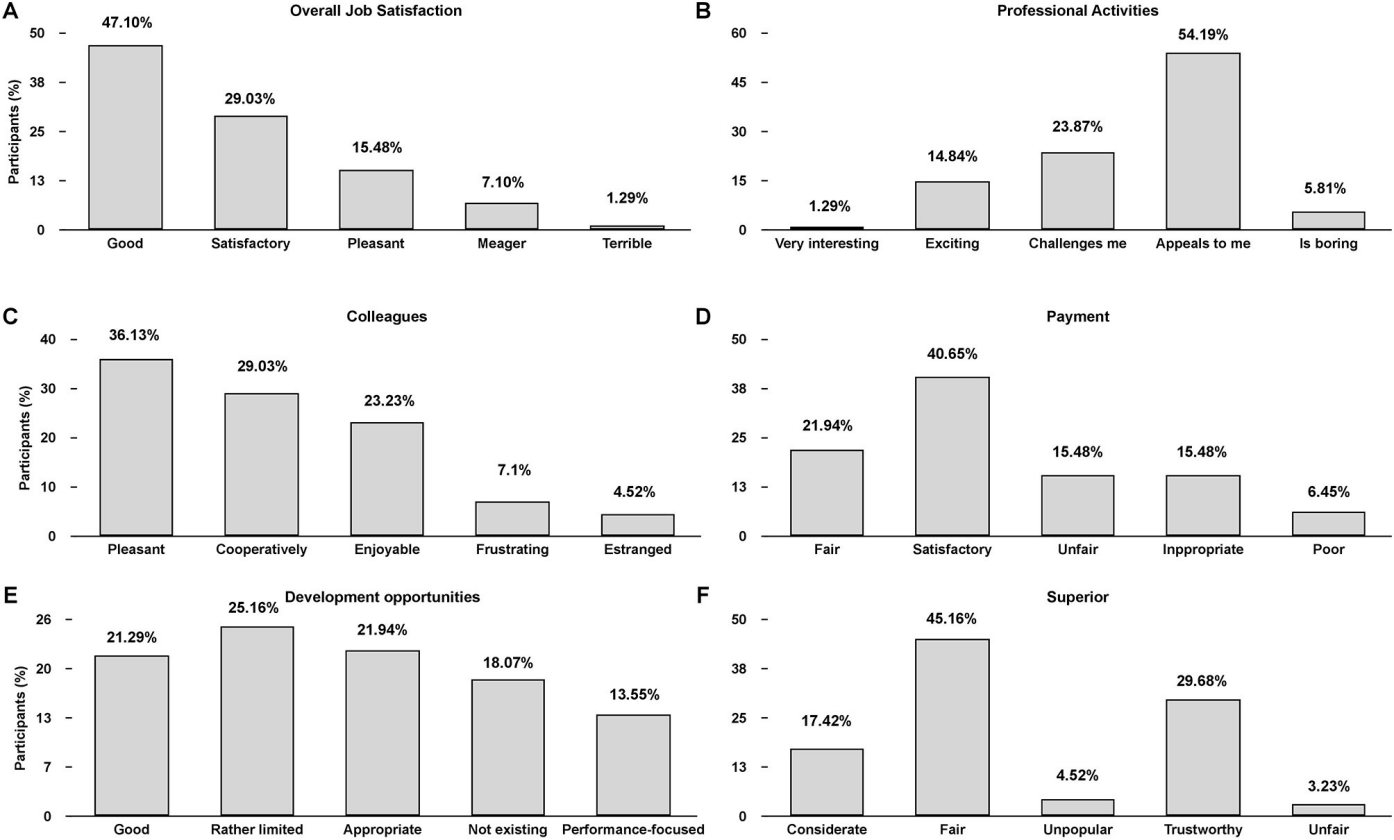
Assessment of Physician Assistants job satisfaction in Germany (percentage response frequencies, *n* = 169). Overall job satisfaction **(A)** and different facets of job satisfaction were assessed: Professional activities **(B)**, colleagues **(C)**, payment **(D)**, development opportunities **(E)**, and superior **(F)**. Only one answer per item **(A–F)** could be selected.

Job satisfaction was assessed using the KAFA, which allows only one answer per item. The overall job satisfaction of participating PAs was good (47.1%), satisfactory (29.0%), and pleasant (15.5%; Figure 1A). Few PAs rated their general job satisfaction with meager (7.1%) or terrible (1.3%). Regarding the professional activities (Figure 1B), most participating PAs were rated as appealing to them (54.2%), followed by being challenging (23.9%), and exciting (14.8%). Colleagues (Figure 1C) were mainly seen as pleasant (36.13%), cooperative (29.0%), and enjoyable (23.2%). A total of 40.7% of study participants were satisfied with the payment (Figure 1D). Only 15.5 and 6.5% rated their payment as unfair or poor, respectively. Development opportunities (Figure 1E) for participating PAs indicated a broad distribution, ranging from 21.3% stating good, 25.2% as rather limited, 22.0% as appropriate, and 18.1% as not existing. An additional 13.6% mentioned their development opportunities being performance focused. Superiors (Figure 1F) of most participating PAs were assessed as fair (45.2%) and trustworthy (29.7%).

### Job satisfaction of PAs working in different medical areas

The overall job satisfaction was not much different between male and female PAs or PAs working in rural or urban regions (Table 3). Moreover, overall job satisfaction seemed to be rather independent of the net income per month since all pay grades rated their overall job satisfaction as good. Calculation of the job satisfaction per medical working area demonstrated that the majority of PAs working in surgery, internal medicine, emergency, geriatrics, and oncology rated their job satisfaction as good. The small subgroup of participating PAs working in oncology demonstrated the highest job satisfaction, followed by PAs working in geriatrics and emergency care. Working in internal medicine or surgery did not affect job satisfaction compared to colleagues not working in these fields, but PAs working in orthopedics or neurology rated their job satisfaction as lower. Most PAs in neurology rated their job as only satisfactory. Only a few PAs declared terrible job satisfaction, with all of them working in surgery.

**Table 3:** Overall job satisfaction in German Physician assistants by gender, region, net income per month, and medical working area.

	**Overall job satisfaction**				
	**Good**	**Pleasant**	**Satis-factory**	**Meager**	**Terrible**
**Gender**					
Male (*n* = 21)	47.6	9.5	28.6	14.3	0.0
Female (*n* = 122)	45.1	17.2	29.5	6.6	1.6
**Region**					
Rural (*n* = 54)	40.7	18.5	33.3	5.6	1.9
Urban (*n* = 89)	48.3	14.6	27.0	9.0	1.1
**Net income per month**					
≤ 999 EUR (*n* = 3)	0.0	0.0	100.0	0.0	0.0
1,000–1,499 EUR (*n* = 6)	83.3	0.0	16.7	0.0	0.0
1,500–1,999 EUR (*n* = 14)	50.0	21.4	14.3	14.3	0.0
2,000–2,499 EUR (*n* = 74)	37.8	16.2	33.8	10.8	1.4
≥2,500 EUR (*n* = 46)	54.3	17.4	23.9	2.1	2.1
**Medical area**					
Surgery (*n* = 33)	48.5	18.2	18.2	9.1	6.1
Internal medicine (*n* = 29)	48.3	17.2	20.7	13.8	0.0
Orthopedics (*n* = 25)	40.0	12.0	40.0	8.0	0.0
Emergency (*n* = 11)	54.5	9.1	27.3	9.1	0.0
Geriatrics (*n* = 8)	62.5	0.0	25.0	12.1	0.0
Neurology (*n* = 7)	28.6	14.3	57.1	0.0	0.0
Oncology (*n* = 4)	100.0	0.0	0.0	0.0	0.0
Others (*n* = 40)	47.5	17.5	32.5	2.5	0.0

The assessments of job satisfaction are given as a percentage of the PAs (n = total number) in the different subgroups, working (yes) or not working (no) in different medical areas.

### Depression-anxiety-stress scale of PAs

Analysis of the levels of depression, anxiety, and stress of participating PAs was determined using the Depression-Anxiety-Stress Scale (DASS-21) (45), and the results are shown in Table 4. The overall study population indicated depression, anxiety, and stress scores of 6.2 (SD 7.2), 4.5 (SD 6.0), and 9.4 (SD 7.4) on the DASS-21 subscales, respectively. With regard to the cutoff values (cutoff value of 10 for depression, 8 for anxiety, or 15 for stress, for which an increased expression of these characteristics can be assumed), there was no increased depression, anxiety, or stress score observed within the group of participating PAs. Taking a closer look at the subgroups, depression score levels exceeded the cutoff value of PAs working in neurology or geriatrics units, having a net income of 1,500–1,999 EUR or assessing their overall job satisfaction as meager. The latter PA subgroup also demonstrated an increased scale score for stress. However, these findings had to be taken carefully as the mentioned subgroups were relatively small.

**Table 4:** DASS-21 subscale scores [mean and standard deviation (SD)] of German PAs related to sociodemographic and work-related factors on mental health.

**Variables**		**Depression mean (SD)**	**Anxiety mean (SD)**	**Stress mean (SD)**
Gender	Male (*n* = 21)	7.52 (6.95)	**2.29 (3.36)** ^*^	8.86 (6.83)
	Female (*n* = 122)	6.00 (7.26)	**4.82 (6.29)** ^*^	9.46 (7.56)
Region	Rural (*n* = 54)	5.20 (6.01)	**3.26 (5.43)** ^*^	8.19 (6.54)
	Urban (*n* = 89)	6.79 (7.84)	**5.17 (6.26)** ^*^	10.09 (7.88)
Medical area	Internal medicine—no (*n* = 114)	6.40 (7.51)	4.83 (6.37)	9.51 (7.71)
	Internal medicine—yes (*n* = 29)	5.52 (5.99)	2.97 (4.06)	8.83 (6.36)
	Orthopedics—no (*n* = 118)	6.10 (6.96)	**3.90 (5.00)** ^*^	9.29 (7.11)
	Orthopedics—yes (*n* = 25)	6.80 (8.45)	**7.04 (9.13)** ^*^	9.76 (8.99)
	Surgery—no (*n* = 110)	5.89 (7.39)	4.47 (6.49)	9.44 (7.93)
	Surgery—yes (*n* = 33)	7.33 (6.59)	4.36 (4.14)	9.15 (5.57)
	Emergency medicine—no (*n* = 132)	6.12 (7.25)	4.49 (6.20)	9.53 (7.53)
	Emergency medicine—yes (*n* = 11)	7.46 (6.99)	4.00 (3.10)	7.46 (6.20)
	Neurology—no (*n* = 136)	6.03 (7.05)	4.50 (6.10)	9.31 (7.28)
	Neurology—yes (*n* = 7)	10.00 (9.87)	3.43 (3.95)	10.57 (10.63)
	Geriatrics—no (135)	5.91 (6.87)	4.27 (5.92)	9.05 (7.11)
	Geriatrics—yes (*n* = 8)	11.50 (10.89)	7.50 (7.15)	14.75 (10.90)
Responsibilities	Anamnesis—no (*n* = 28)	7.00 (6.57)	**6.07 (5.35)** ^*^	**11.43 (7.01)** ^*^
	Anamnesis—yes (*n* = 115)	6.04 (7.38)	**4.05 (6.12)** ^*^	**8.87 (7.48)** ^*^
	Diagnostic execution—no (*n* = 41)	5.71 (6.40)	4.44 (4.35)	9.12 (6.15)
	Diagnostic execution—yes (*n* = 102)	6.43 (7.54)	4.45 (6.58)	9.47 (7.92)
	Diagnostic analysis—no (*n* = 62)	7.13 (7.51)	**5.65 (6.99)** ^*^	10.32 (8.03)
	Diagnostic analysis—yes (*n* = 81)	5.53 (6.95)	**3.53 (4.99)** ^*^	8.64 (6.91)
	Therapy proposal—no (*n* = 62)	6.81 (7.57)	5.13 (6.75)	9.74 (7.56)
	Therapy proposal—yes (*n* = 81)	5.78 (6.95)	3.93 (5.36)	9.09 (7.38)
	Therapy execution—no (*n* = 47)	6.55 (7.46)	4.98 (6.74)	9.49 (8.16)
	Therapy execution—yes (*n* = 96)	6.06 (7.13)	4.19 (5.64)	9.32 (7.10)
	Nursing—no (*n* = 123)	6.59 (7.46)	4.57 (6.34)	9.74 (7.71)
	Nursing—yes (*n* = 20)	4.00 (5.07)	3.70 (3.39)	7.10 (5.05)
	Patient education—no (*n* = 54)	5.22 (6.06)	4.59 (4.86)	9.00 (7.19)
	Patient education—yes (*n* = 89)	6.83 (7.80)	4.36 (6.64)	9.60 (7.61)
	Prevention measures—no (*n* = 85)	6.94 (7.92)	5.06 (6.78)	9.84 (8.24)
	Prevention measures—yes (*n* = 58)	5.17 (5.95)	3.55 (4.57)	8.69 (6.07)
	Follow-up examination—no (*n* = 85)	5.91 (6.46)	3.93 (4.46)	8.78 (6.84)
	Follow-up examination—yes (*n* = 58)	6.69 (8.24)	5.21 (7.73)	10.24 (8.22)
	Documentation—no (*n* = 11)	5.82 (6.42)	4.73 (5.39)	9.46 (5.45)
	Documentation—yes (*n* = 132)	6.26 (7.30)	4.42 (6.08)	9.36 (7.59)
	Surgery participation—no (*n* = 69)	7.30 (7.65)	4.38 (5.76)	10.35 (8.00)
	Surgery participation—yes (*n* = 74)	5.22 (6.69)	4.51 (6.27)	8.56 (6.80)
	Team coordination—no (*n* = 76)	6.42 (6.88)	4.61 (6.05)	9.87 (7.49)
	Team coordination—yes (*n* = 67)	6.00 (7.63)	4.27 (6.01)	8.81 (7.39)
Net income per month^**#**^	≤ 999 EUR (*n* = 3)	–	–	–
	1,000–1,499 EUR (*n* = 6)	3.67 (4.80)	2.00 (4.00)	8.33 (4.63)
Job satisfaction^#^	1,500–1,999 EUR (*n* = 14)	10.43 (10.23)	5.57 (7.97)	11.29 (8.58)
	2,000–2,499 EUR (*n* = 74)	5.97 (5.79)	5.11 (5.57)	9.35 (6.83)
	≥2,500 EUR (*n* = 46)	5.65 (8.13)	3.44 (6.31)	9.09 (8.43)
	Good (*n* = 65)	**4.22 (6.15)** ^***^	**3.29 (4.28)** ^*^	**8.25 (6.54)** ^*^
	Pleasant (*n* = 23)	**7.04 (7.38)** ^***^	**4.87 (5.93)** ^*^	**9.22 (9.22)** ^*^
	Satisfactory (*n* = 42)	**6.43 (7.39)** ^***^	**5.38 (8.26)** ^*^	**9.33 (7.57)** ^*^
	Meager (*n* = 11)	13.64 (6.12) ^***^	**6.91 (4.42)** ^*^	15.27 (7.96) ^*^
	Terrible (*n* = 2)	–	–	–

The p-values of 0.05 or less were considered statistically significant and marked (bold, ^*^p ≤ 0.05; ^**^p ≤ 0.01; ^***^p ≤ 0.001; ^#^Kruskal–Wallis Test). Scale scores exeeding cutoff scores fpr DASS-21 were underlined.

Scores for depression, anxiety, and stress were significantly dependent on job satisfaction (*p* ≤ 0.001, *p* ≤ 0.05, and *p* ≤ 0.05, respectively). *Post hoc* analysis revealed that those who rated their job as meager had a significantly different depression score compared to those rating good (*p*bonf ≤ 0.001) and pleasant (*p*bonf = 0.029). In addition, anxiety (*p* = 0.031) and stress levels (*p* = 0.036) also significantly varied depending on job satisfaction. *Post hoc* analysis of the subscale scores revealed that those who rated their job as meager indicated significantly higher levels compared to those rating good (anxiety: pbonf = 0.016/stress: pbonf = 0.025). The DASS-21 subscale scores were not significantly related to net income per month.

Based on the Mann–Whitney *U*-Test, analysis of sociodemographic effects on DASS-21 scales revealed that female PAs indicated a significantly higher anxiety score compared to male PAs (*p* ≤ 0.05), and PAs working in an urban environment indicated a significantly higher anxiety score compared to PAs in a rural setting (*p* ≤ 0.05). In addition, PAs working in orthopedic wards revealed significantly higher anxiety scores (*p* ≤ 0.05). The anxiety and stress levels were significantly increased in the subgroup of PAs who were not allowed to conduct anamnesis (both *p* ≤ 0.05). Furthermore, anxiety levels were also significantly increased in the group of participating PAs who were not allowed to perform diagnostic analysis (*p* ≤ 0.05).

## Discussion

This cross-sectional survey study characterized currently working PAs in Germany, documented the responsibilities, empowerments, and fields of activity for the first time, and demonstrated a relationship between job satisfaction, medical working areas, and depression, anxiety, and stress. Scores for depression, anxiety, and stress were significantly negatively correlated to overall job satisfaction and determined by responsibilities and medical working area.

In Germany, currently working PAs are mainly female, about 30 years old on average, and single or living with partner as usual for this age group (48). 62.7% of the respondents are working in an urban region. The net salary of more than 50% of the respondent PAs is 2,000–2,499 EUR, and about one-third earned more than 2,500 EUR per month, slightly more than the average salary in Germany in 2021 of 4.100 euros gross per month (49), which is about 2.500 euros net per month depending on tax class. PAs are mainly working in surgery, internal medicine, and emergency, but also in orthopedics. Only a few are currently deployed in geriatrics, neurology, or oncology. They share nearly the same profile of authorizations and responsibilities with differences according to medical working area (documentation, anamnesis, and diagnostic services were mentioned most frequently) and rate their overall job satisfaction mainly as good. PAs working in oncology demonstrated the highest overall job satisfaction, followed by PAs working in geriatrics and emergency care.

PA's scores for depression, anxiety, and stress were significantly negatively correlated to overall job satisfaction. Moreover, scores for depression and stress exceed critical cutoff values of PAs with meager overall job satisfaction, highlighting the importance of taking a closer look at the different facets of job satisfaction and the underlying needs and causes. Moreover, in other research, job dissatisfaction and symptoms of burnout were correlated with age and years of practice (35, 50). Thus, one has to take a closer look at the first signs of job dissatisfaction and correlated mental state so as to not underestimate the risks of the young group of PAs in Germany.

Indeed, even slight gradations of high job satisfaction may predict psychological stresses. A survey of PAs in Minnesota demonstrated that, despite high levels of career and job satisfaction, PAs reported moderate levels of burnout, particularly women in primary care (22). In fact, in this study, we also found PA subgroups with increased scores for depression, anxiety, and stress, although PAs declared their overall job satisfaction as good. We also identified significantly increased anxiety scores of female PAs in comparison to male PAs as well as of PAs working in an urban environment, which was the case for a majority of the respondent PAs in this study. It was not clear why urban PAs reached higher anxiety scores than their rural colleagues, especially since previous studies demonstrated that working conditions were worse in the city. For example, in Germany, rural general practitioners worked significantly more hours per week than their urban colleagues (51). PAs working in rural areas in the U.S. also reported an insufficient physician density, a lack of young recruits in primary care, and a resulting increased workload, whereas their urban colleagues reported a high physician density in urban areas, associated with high competition between general practitioners, a high fluctuation of patients, and a low status of general practitioners (52). In addition, it was assumed that rural PAs possessed a larger scope of practice than urban PAs (53). Since job experience was known to reduce psychological distress and burnout (31, 54), the increased anxiety score of urban PAs in Germany might be a consequence of a lower scope of practice, less responsibilities, and less job experience. Indeed, in this study, anxiety as well as stress scores were significantly reduced in the group of PAs with authorization to take anamnesis, and the anxiety score was significantly reduced in the group of PAs with authorization for diagnostic analysis.

Anamnesis and diagnostic services were the responsibilities of nearly all respondent PAs, as well as documentation. Depending on the medical working area of German PAs, responsibilities were slightly different, reaching from intervention/counseling and treatment suggestions to medical report/diagnostic analysis, patient information, team coordination, prevention and instruction, surgery participation, after-care, other responsibilities, nursing activities, and telemedical care (listed according to their percentage frequency). None of the respondent PAs did home visits. In summary, PAs working in emergency care claimed to be those with the most responsibilities, followed by PAs in orthopedics and surgery. However, PAs working in areas with the highest authorization levels (emergency, orthopedics, and surgery) did not necessarily rate their job satisfaction higher.

With regard to depression, anxiety, and stress, no consistent correlation to the medical working area or the number of responsibilities could be detected. Although other studies have shown that nearly all PAs were working in areas with high burnout prevalence (35), in this study, PAs working in orthopedics, neurology, or geriatrics rated their DASS scores as the worst. Depression scores of PAs working in orthopedics were significantly increased in comparison to those of PAs working in other medical areas and PAs working in neurology or geriatrics have depression scores that exceeded the critical cutoff value of 10. Previous studies identified that high workload and a low level of job control/loss of autonomy were associated with a high prevalence of burnout and low rates of job satisfaction among healthcare workers in all three professions (42, 50, 55). Since these subgroups of PAs rated their overall job satisfaction differently and demonstrated different sets of responsibilities, no common cause for increased depression or anxiety could be inferred.

Depression scores also exceeded the cutoff value in PAs with a net income per month of 1,500–1,999 EUR. The number of PAs in these groups was small, but a positive association between financial stress, which might be triggered by income below average here, and depression had already been found in different countries (56). Indeed, most respondent PAs rated their payment more satisfactory than fair, even one-third of PAs rated net income as unfair, inappropriate, or poor. Since PAs declared their overall job satisfaction as good independent of the net income per month and a poor rating of the facet payment, this facet did not have a high impact on the overall rating of job satisfaction. Concerning the satisfying role of payment, previous studies have demonstrated that, although income was often attested to have a strong motivating effect (57), empirical studies only detected moderate correlations between income and job satisfaction (58), and job dissatisfaction with promotion and training opportunities were found to have a stronger impact than workload or pay (7).

Respondent PAs evaluated the facets of job satisfaction as “colleagues and supervisors” very positively, whereas professional activities were rated rather average and development opportunities even worse. Thus, PA's assessment of good overall job satisfaction seemed to be mainly influenced by the good assessment of colleagues and supervisors. This facet seems to be weighted differently since the poorer rating of the other facets cannot explain the overall positive rating. The importance of the social environment to wellbeing and job satisfaction has already been demonstrated in surveys with physicians or physician assistants in the U.S. For example, previous studies demonstrated that PA's overall job satisfaction was associated with satisfaction with one's supervising physician and satisfaction with the community and autonomy (59, 60), and retention in the job (considered a proxy for satisfaction) was closely linked to confidence in clinical abilities and community embeddedness (61). It was shown in other research that leadership quality explained almost half the variation in physician satisfaction scores in physicians with high satisfaction ratings hboxcitepbib24,bib35. Moreover, burnout rates were higher in physicians who rated their leaders unfavorably. Teamwork was among the factors that PAs felt contributed to their satisfaction (35), and team-based practice has been shown to cultivate an environment that reduces symptoms of burnout in primary care (62).

In Germany, PAs are highly dependent on the delegating physician. This might explain the importance of a good supervisor on PA's job satisfaction. The young PA profession was still highly regimented in Germany. PAs should relieve the medical team by taking over delegable tasks (2), and their professional activities were regulated by laws permitting the delegation of medical tasks to non-physician health professionals (63, 64). The scope of practice was determined by a delegation from the supervising physician and varies between practice settings (1, 64, 65). Thus, it was not surprising that PAs face challenges in balancing autonomy and dependence, especially since PAs were highly educated in Germany and patients also felt comfortable seeing a PA instead of a doctor (64). These challenges were of particular importance for job satisfaction and mental health since it has been demonstrated that PA's overall job satisfaction was associated with autonomy (59, 60). In addition, the main areas of application for PAs hardly changed in the last 10 years, where they were already generally deployed within a surgical, internal medicine, and emergency medicine setting (1), suggesting that professional activities and responsibilities remained almost the same. These facts might explain why more than half of the respondents in this study evaluated the professional activities only as appealing to them, and even 5% declared them as boring.

PAs rated development opportunities the worst and least consistently, and the assessments range in roughly equal parts from good to not existing. Indeed, in international comparison, development opportunities in academic settings were low in Germany. Studying PA was a bachelor's degree, whereas in most other countries, PA was a master's degree, which typically follows a bachelor's degree, e.g., in nursing (2). There are only a few options for completing a PA master's degree in Germany, although the desire for a master's program was becoming apparent years ago (1). Moreover, many PAs completed training in a healthcare profession before going to college (2) and training/working as a PA might not be financially beneficial for experienced healthcare workers (64). Furthermore, in Germany, patients often did not understand the PA title or role because of the limited awareness of the PA profession in the medical field and the public sphere. Thus, the professional recognition of PAs in Germany had to be improved, especially since studies with PAs in the U.S. demonstrated that general misunderstanding of the PA role (role ambiguity) resulted in dissatisfaction (35).

Although medical professionals increasingly viewed PAs very positively, there are still some concerns about PAs expressed in Germany (64). In comparison to the U.S., where the PA's job profile and educational programs are well-established (66), PAs were still new in Germany. In the U.S., PAs have more duties and responsibilities depending on the medical working area, job experience, and state law (67). Within the physician–PA relationship, PAs exercise autonomy in medical decision-making and provide a broad range of diagnostic and therapeutic services. A PA's practice might also include education, research, and administrative servicesTeaching was identified as a protective factor against burnout among emergency medicine PAs in the U.S. and may also serve as a protective measure for clinically practicing PAs (68). Thus, PAs in the U.S. have more authority/autonomy and a larger job profile than PAs in Germany. They also rated their overall job satisfaction high, but about one in five PAs in the U.S. indicated an intent to reduce clinical hours within the next year, and one in three Pas indicated an intent to leave their current clinical practice within the next 2 years (69). Salary, autonomy, job resources, advancement opportunities, and quality of relationships with collaborating physicians and other team members were associated with job satisfaction among PAs in the U.S. (69). We were able to identify almost the same factors in this study determining the job satisfaction of German PAs. Thus, not only in Germany, the factors of job satisfaction might assist policymakers and health administrators in creating welcoming professional employment environments (60).

## Conclusion

PAs in Germany were happy in their jobs, mainly because of their colleagues and supervisors, and demonstrated an overall good status for depression, anxiety, and stress, although women and rural PAs need special support regarding the prevention of anxiety. PA's scope of practice is dependent on the medical working area. Payment, professional activities, and even more development opportunities need to be more appealing. To further establish the job profile in Germany and to retain PAs in their job, the leadership qualities of supervisors should be trained and maintained, team cohesion should be promoted, and clear role allocation should be ensured. Better visibility and acceptance of the job profile could be achieved through clearer national regulations and awareness campaigns. The success of the professional activities and responsibilities of the PAs in everyday life should be observed and reliably evaluated with regard to patient safety and satisfaction to possibly increase the scope of the autonomously executable activities with years of professional experience, after further training and opportunities for advancement.

## Data availability statement

The original contributions presented in the study are included in the article/supplementary material, further inquiries can be directed to the corresponding author.

## Ethics statement

The study was approved by the ethics committee of the HSD University of Applied Sciences, Germany (BEth_54_222). Written informed consent from the participants was not required to participate in this study in accordance with the national legislation and the institutional requirements.

## Author contributions

YT and KK conceived of the presented idea and designed the survey, supervised by LM. LM performed the computations and statistics. KK took the lead in writing the manuscript. All authors discussed the results and contributed to the final manuscript.
